# The Adherent/Invasive Escherichia coli Strain LF82 Invades and Persists in Human Prostate Cell Line RWPE-1, Activating a Strong Inflammatory Response

**DOI:** 10.1128/IAI.00438-16

**Published:** 2016-10-17

**Authors:** Maria P. Conte, Marta Aleandri, Massimiliano Marazzato, Antonietta L. Conte, Cecilia Ambrosi, Mauro Nicoletti, Carlo Zagaglia, Guido Gambara, Fioretta Palombi, Paola De Cesaris, Elio Ziparo, Anna T. Palamara, Anna Riccioli, Catia Longhi

**Affiliations:** a Department of Public Health and Infectious Diseases, Sapienza University, Rome, Italy; b Department of Medical, Oral and Biotechnological Sciences, G. D'Annunzio University, Chieti, Italy; c Center of Space Medicine Berlin, Neuromuscular System, Institute of Anatomy, Charité-Universitätsmedizin Berlin, Berlin, Germany; d Department of Anatomy, Histology, Forensic Medicine and Orthopedics, Unit of Histology and Medical Embryology, Sapienza University, Rome, Italy; e Department of Biotechnological and Applied Clinical Sciences, University of L'Aquila, L'Aquila, Italy; f Department of Public Health and Infectious Diseases, Pasteur Institute Cenci Bolognetti Foundation, Sapienza University, Rome, Italy; g IRCCS, San Raffaele Pisana, Rome, Italy; University of Texas at Austin

## Abstract

Adherent/invasive Escherichia coli (AIEC) strains have recently been receiving increased attention because they are more prevalent and persistent in the intestine of Crohn's disease (CD) patients than in healthy subjects. Since AIEC strains show a high percentage of similarity to extraintestinal pathogenic E. coli (ExPEC), neonatal meningitis-associated E. coli (NMEC), and uropathogenic E. coli (UPEC) strains, here we compared AIEC strain LF82 with a UPEC isolate (strain EC73) to assess whether LF82 would be able to infect prostate cells as an extraintestinal target. The virulence phenotypes of both strains were determined by using the RWPE-1 prostate cell line. The results obtained indicated that LF82 and EC73 are able to adhere to, invade, and survive within prostate epithelial cells. Invasion was confirmed by immunofluorescence and electron microscopy. Moreover, cytochalasin D and colchicine strongly inhibited bacterial uptake of both strains, indicating the involvement of actin microfilaments and microtubules in host cell invasion. Moreover, both strains belong to phylogenetic group B2 and are strong biofilm producers. *In silico* analysis reveals that LF82 shares with UPEC strains several virulence factors: namely, type 1 pili, the group II capsule, the vacuolating autotransporter toxin, four iron uptake systems, and the pathogenic island (PAI). Furthermore, compared to EC73, LF82 induces in RWPE-1 cells a marked increase of phosphorylation of mitogen-activated protein kinases (MAPKs) and of NF-κB already by 5 min postinfection, thus inducing a strong inflammatory response. Our *in vitro* data support the hypothesis that AIEC strains might play a role in prostatitis, and, by exploiting host-cell signaling pathways controlling the innate immune response, likely facilitate bacterial multiplication and dissemination within the male genitourinary tract.

## INTRODUCTION

*E*scherichia coli strains are the most abundant facultative anaerobic bacteria of the normal human gut flora, which include a variety of nonpathogenic commensals as well as a set of pathogenic variants that cause intestinal (intestinal pathogenic E. coli [IPEC]) as well as extraintestinal (extraintestinal pathogenic E. coli [ExPEC]) infections (1). While IPEC strains are obligate intestinal pathogens, ExPEC strains live as commensals in the digestive tract of the host (2). Compared to most commensal E. coli strains, which generally belong to the A and B1 phylogenetic groups, most ExPEC strains belong to the B2 or D groups and express highly diverse virulence factors (3). ExPEC strains have been classified into three major groups based on disease association, comprising uropathogenic E. coli (UPEC), neonatal meningitis-associated E. coli (NMEC), and sepsis-causing E. coli (SEPEC). However, such classification is rather restrictive, since no single virulence factor renders an ExPEC isolate capable of causing site-specific disease and especially because isolates assigned to a specific ExPEC group may infect different anatomic sites (3). Adherent/invasive E. coli (AIEC), a particular E. coli pathotype, has been isolated from patients with Crohn's disease (CD), and several data suggest a role of these strains in the pathogenesis of CD (4). Interestingly, AIEC strains have also been detected in ileal and colonic specimens from healthy subjects, suggesting their classification as pathobionts (5). AIEC strains do not carry virulence genes so far identified among IPEC strains, while analysis of the available complete genomic sequences of different AIEC strains revealed a phylogenetic linkage with ExPEC rather than with IPEC (6) and in particular with the pathotypes associated with urinary tract infections (UTIs) and neonatal meningitis (7). AIEC and ExPEC strains share some phenotypic traits, including the ability to adhere to and invade host cells (8) and the ability to induce an inflammatory response in animal models (9) as well as in polarized intestinal epithelial cells (10). AIEC strain LF82 represents the prototype of AIEC strains and belongs to phylogroup B2, typical of ExPEC (3, 11–13). Adhesion and invasion of intestinal epithelial cells by LF82 require the expression of several virulence determinants such as type 1 pili, several outer membrane proteins (OMPs), and the IbeA invasin (9, 14–16). In particular, it has been shown that FimH, the terminal subunit of type 1 pili, interacts specifically with mannosylated carcinoembryonic antigen-related cell adhesion molecule 6 (CEACAM6), which is overexpressed in ileal CD tissue (15). It has been demonstrated that allelic variation of the *fimH* gene may confer significant advantage in gut colonization and to the virulence of AIEC and ExPEC (9, 17). In the ExPEC uropathogenic E. coli (UPEC) strains, expression of type 1 pili enhances colonization of the urothelial mucosa, promotes biofilm formation and host cell invasion, and induces expression of proinflammatory cytokines (18–21). Moreover, some UPEC strains are able to persist within infected tissues due to their ability to inhibit NF-κB activation, modulate expression and release of proinflammatory cytokines (22–24), and form intracellular bacterial communities, which allows bacteria to resist the host immune response and antibiotic therapy (25–28). On the other hand, it has been shown that AIEC strains are stronger biofilm producers (29) and can subvert the innate immune response (30), allowing them to persistently colonize the intestinal mucosa. Similarly, UPEC strains causing UTIs and prostatitis mainly belong to the B2 phylogenetic group and show a gradient of virulence traits, including a greater tendency to develop biofilm-like structures (31, 32). We have recently demonstrated the ability of as many as 58 UPEC strains to adhere to and invade human prostate cells with high efficiency (33). These findings, together with the carriage of some virulence-associated genes characteristic of ExPEC pathovars, led us to hypothesize that AIEC strains may also infect body sites other than the intestine. To explore this point, in this article, we compare the behavior of AIEC strain LF82 to that of an E. coli strain (EC73) isolated from a subject with recurrent UTIs. In the present study, the ability of strains LF82 and EC73 to adhere to, invade, survive intracellularly, and induce inflammation was studied by experimentally infecting the human prostate RWPE-1 cell line. The carriage of some virulence genes was also determined, and alteration of signal transduction pathways and release of proinflammatory cytokines interleukin-6 (IL-6) and IL-8 were studied. The results obtained clearly indicate that, like EC73, LF82 is able to invade and to survive within prostate cells. Interestingly, differently from EC73, LF82 is also able to elicit a strong inflammatory response. In conclusion, our data led us to suggest that AIEC strains have the potential to invade prostate cells, potentially causing prostatitis.

## MATERIALS AND METHODS

### Bacterial strains and culture conditions.

The prototype adherent/invasive AIEC strain LF82 (a gift of Arlette Darfeuille-Michaud, Université of Auvergne, France) was isolated from a chronic ileal lesion from a CD patient (11). E. coli EC73 is one of three UPEC strains (EC71, EC72, and EC73) collected over a 2-year time span from a 57-year-old male suffering from recurrent UTIs. E. coli K-12 strain MG1655 was used as a control in adhesion-invasion assays, and enteroinvasive E. coli (EIEC) strain HN280 was used as a prototype intestinal pathogen in the invasion assay. All strains were grown on brain heart infusion (BHI) broth (Oxoid, Rome, Italy) and on Trypticase soy agar (TSA [Oxoid]) overnight at 37°C.

### Cell lines and cell culture.

The RWPE-1 cell line (derived from prostate epithelial cells isolated from the peripheral zone of a nonneoplastic human prostate and immortalized with human papillomavirus 18) were purchased from ATCC (Manassas, VA). These cells mimic normal prostate epithelial cell behavior in their response to growth factors and in the expression of prostate-specific antigen (PSA) and androgen receptor. RWPE-1 cells were maintained in a humidified atmosphere of 5% CO_2_ at 37°C in 1% in keratinocyte serum-free medium (K-SFM) (Gibco, Life Technologies) supplemented with 0.05 mg/ml bovine pituitary extract (BPE), 5 ng/ml human recombinant epidermal growth factor (EGF), and 20 μg/ml gentamicin. Human Caco-2 (ATCC HTB-37) cells were grown in minimum essential medium (MEM [Euroclone, Milan, Italy]). HEp-2 cells (ATCC CCL-23) were maintained in Eagle's minimal essential medium (EMEM [Sigma, Italy]). Both lines were supplemented with 5% heat-inactivated fetal calf serum (FCS [Euroclone, Italy]) and 1% penicillin-streptomycin.

### PFGE typing.

The genetic relationship among the three UPEC isolates was determined by pulsed-field gel electrophoresis (PFGE) typing as previously described (34). The Dice coefficient of similarity was calculated, and the unweighted pair group method with arithmetic averages (UPGMA) was used for cluster analysis. Strains were considered identical if no fragment differences occurred.

### Biofilm assay.

Biofilm formation was assayed as previously described (35). ATCC E. coli strain 25922 and UPEC strain 16 (33), were used as biofilm positive and negative controls, respectively. After 24 h of incubation at 30°C, wells were extensively washed with phosphate-buffered saline (PBS), and attached bacteria were stained with crystal violet (0.1% [vol/vol]) for 15 min. The stain was released with 150 μl of 80% (vol/vol) ethanol. Biofilms were quantified by measuring the absorbance at λ 595 nm (*A*_595_) with a microplate reader (Tecan Sunrise, X-fluor). According to their absorbance, isolates were defined as strong (*A*_595_, >0.7), medium (0.6 > *A*_595_ > 0.4), or weak (0.3 > *A*_595_ > 0.1) biofilm producers or non-biofilm producers (*A*_595_, <0.1).

### Phylogenetic PCR grouping.

Phylogenetic analysis of E. coli strains was carried out by multiplex PCR, as previously described (36). Whole-DNA bacterial extracts were prepared using Qiagen DNA extraction kit (Qiagen, Italy). Amplifications were performed with a Perkin-Elmer GeneAmp 9600 thermal cycler, and amplicons were separated by electrophoresis in 2% agarose. AIEC strain LF82 was included as an internal control (29).

### *In silico* virulence genotyping.

The presence of 26 virulence-associated factors of ExPEC was determined by *in silico* analysis. Gene sequences were taken from GenBank and tested against the whole genomes of the LF82, CFT073, and UTI89 strains using the BLASTn algorithm included in BLAST+ v.2.4.0. These include genes associated with adhesins (*papP*, *sfaS*, *focF1C*, *fim* type I, *afa*, *nfaE*, and *gafD*), capsule synthesis (*kpsMTII*, *kpsMTIII*, and *rfc*), iron acquisition (*ent*, *iro*, *chu*, *sit*, *fyuA*, and *iut*A), toxins (*cnf1*, *cdt*, *cva*C, *hlyA*, *vat*, and *sat*), the pathogenicity-associated island (PAI), invasin (*ibeA*), serum resistance (*traT*), and immune evasion (*tcpC*). Hits presenting a query coverage of ≥90% and a pairwise identity percentage (percentage of pairwise residues that are identical in the alignment, including gap versus non-gap residues but excluding gap versus gap residues) of ≥85% were considered positive. For strain EC73, the presence of virulence genes was tested on genomic data produced by next-generation sequencing. Briefly, 2 ml of an overnight bacterial culture of EC73 was used for total genomic DNA extraction using the GenElute bacterial genomic DNA kit (Sigma-Aldrich, Italy) according to the manufacturer's instructions. DNA final elution was performed in water. DNA was sequenced using 250-bp paired-end reads on the Illumine MiSeq system by Bio-Fab Research (Rome, Italy), producing about 7 million raw sequence reads in the Fastq format, corresponding to about 300-fold estimated genome coverage. Raw data were imported in Geneious v.7.1.9 (Biomatters, Inc., USA) and trimmed in order to remove index sequences, adapter sequences, and poor-quality sequenced bases. Filtered data were mapped to reference sequences using Bowtie2 v.2.2.9 (37). Hits presenting a query coverage of ≥95% were considered positive.

### Adhesion, invasion, intracellular survival, and multiplication assays.

Adhesiveness of E. coli strains to cultured RWPE-1 and HEP-2 cell monolayers was assayed using standard protocols (34). Bacteria were considered adherent when the mean adhesion index (no. of adherent bacteria/initial inoculum) was ≥0.8%. To assess the role of type 1 pili in adhesion, assays were also carried out in the presence of 0.5% (vol/vol) d-mannose (34, 35, 38). Cell invasion was assayed by infecting cultured RWPE-1, Caco-2, and HEp-2 cell monolayers. Twenty-four-well tissue culture plates were seeded with cells (1 × 10^5^ cells/well) and incubated at 37°C in 5% CO_2_ for 48 h. Cell monolayers were washed and infected with diluted bacterial suspensions (multiplicity of infection [MOI] of 10) in a 0.5-ml volume of cell culture medium devoid of antibiotics. Bacteria were centrifuged onto cell monolayers at 500 × *g* for 2.5 min and incubated for 2 h at 37°C. Two hours postinfection, infected monolayers were washed and incubated for 60 min in growth medium containing gentamicin (100 μg/ml [Fisher Scientific]). In survival and multiplication assays, after the incubation time, medium containing 50 μg/ml gentamicin was added, and multiplication was evaluated at 6, 12, and 24 h postinfection. A strain was considered invasive when the ratio between the number of intracellular bacteria and the initial inoculum was ≥0.1%. Bacteria were considered able to survive when the number of intracellular bacteria recovered at different time points was comparable to that recovered 3 h postinfection (100%). Strains were considered able to replicate intracellularly when the ratio was ≥200%. All assays were performed in triplicate.

### Effect of eukaryotic cytoskeletal inhibitors.

Cell monolayers were preincubated for 30 min prior to the invasion assay in cell culture medium devoid of antibiotics with 1 μg/ml cytochalasin D or 0.5 μg/ml colchicine (Sigma). The inhibitors were present throughout the 2-h bacterial infection period. The inhibitory effect of each inhibitor on bacterial uptake was evaluated against control assays without inhibitors, which were defined as representing 100% of bacterial uptake. All of the assays were performed at least three times in separate experiments.

### Immunofluorescence labeling.

RWPE-1 cell monolayers were infected with E. coli strains (MOI of 10). Cells were washed and incubated for 30 min at 37°C with goat anti-E. coli antibody. After being washed in PBS, cells were incubated with rabbit anti-goat Alexa Fluor 564 (red) diluted 1:500 (Invitrogen). Since this incubation occurred while the plasma membrane was still intact, antibodies only interacted with extracellular bacteria. Samples were then extensively washed with PBS, fixed for 10 min in a solution of 4% paraformaldehyde, and permeabilized with a solution of 0.1% Triton X-100 for 5 min. Cells were washed with PBS and blocked for 45 min with 3% milk in PBS, followed by incubation with goat anti-E. coli antibody and rabbit anti-goat Alexa Fluor 488 (green) diluted 1:500 (Invitrogen) to label intracellular bacteria. Under these experimental conditions, the intracellular bacteria were stained green, whereas extracellular bacteria were orange/yellow (25). Nuclei were stained with 4′,6-diamidino-2-phenylindole (DAPI [Molecular Probes]). Images were acquired by a Leica DM5000B microscope equipped with the Digital FireWire color and black and white camera systems Leica DFX350 and DFX300, respectively, and processed using the Leica Application Suite 2.7.0.R1 software (Leica).

### TEM.

For transmission electron microscopy (TEM), RWPE-1 cells infected with the LF82 and EC73 strains as described above were detached by light trypsinization, yielding densely packed pellets of sphericized cells. These were fixed overnight in cold 2.5% glutaraldehyde in 0.1 M cacodylate buffer, postfixed in 2% osmium tetroxide for 2 h, and then treated for 30 min with 1% tannic acid in 0.05 M cacodylate buffer, dehydrated in ethanol, and processed for Epon embedding. Infected cells were identified in thin toluidine blue-stained sections. Ultrathin sections were contrasted in lead hydroxide and analyzed in a Hitachi 7000 transmission electron microscope.

### SDS-PAGE and Western blot analysis.

Infected RWPE-1 cell monolayers were lysed 5 min, 30 min, and 24 h postinfection as previously described (39). Immunoblot analysis was done with the following antibodies: polyclonal anti-phospho- and anti-ERK1/2 and anti-phospho- and anti-p38 from Cell Signaling and monoclonal antibodies anti-phospho- and anti-JNK1/2 and anti-phospho- and anti-p65 from Santa Cruz, as well as anti-mouse and anti-rabbit secondary antibodies conjugated to horseradish peroxidase from Bio-Rad. The levels of phosphorylated proteins were quantified by densitometry (ImageJ software).

### Cytokine release.

RWPE-1 cell monolayers were infected with E. coli strains (MOI of 10) and incubated at 37°C as described above. Three hours and 24 h postinfection, supernatants were collected and processed for human IL-6 and IL-8 quantification by sandwich enzyme-linked immunosorbent assay (ELISA) Max Deluxe sets (BioLegend, San Diego, CA), following the manufacturer's instructions.

### Statistical analyses.

One-way and repeated-measures analyses of variance (ANOVA) followed by *post hoc* Student's unpaired and paired *t* tests, as needed, were used to asses statistical significance. In all cases, a *P* value of ≤0.05 was considered statistically significant.

## RESULTS

### UPEC isolates EC71, EC72, and EC73 are the same clone.

PFGE analysis of the three UPEC isolates (EC71, EC72 and EC73) showed 100% similarity. E. coli strain EC73 was chosen for further studies.

### Strain LF82 shares with EC73, CFT073, and UTI89 some important virulence factors.

Four main phylogenetic groups (A, B1, B2, and D) characterize the E. coli population. In general virulent ExPEC strains such as AIEC strains mainly belong to groups B2 and D. Furthermore, biofilm production represents an important virulence factor that promotes bacterial growth and persistence at the site of infection and protects bacteria from the host immune response and antimicrobials. As shown in Table 1, strains LF82 and EC73, like CFT073 and UTI89, belong to phylogenetic group B2 and were strong biofilm producers. The *in silico* analysis reveals that LF82 shares with UPEC strains some virulence factors. (i) The first factor is represented by the type 1 group of genes (*fim*). Type 1 pili are a key factor for LF82 adhesion/invasion of intestinal epithelial cells and are essential for successful UPEC adhesion/invasion of the urinary tract epithelial cells. (ii) The factor group II capsule (*kpsMTII*), produced by the majority of the ExPEC strains, has been shown to be essential for the development of UTI and to protect bacteria against complement-mediated killing. (iii) Another factor is the vacuolating autotransporter toxin (Vat), known to contribute to UPEC systemic infections. (iv) Finally, LF82 shares four of six genes of the iron uptake systems investigated: *ent* which encodes siderophore enterobactin, *chu*, which encodes heme transport system, *sit*, which encodes a permease involved in the uptake of iron and manganese, and *fyuA*, which encodes the yersiniabactin receptor, indicating that multiple iron acquisition systems are required to survive and grow within infected tissues.

**TABLE 1:** *In silico* analysis of virulence factors typical of UPEC strains

Factor type or characteristic	Gene	Result for strain:			
		EC73	LF82	CFT073	UTI89
Adhesin	*papP*	+	−	+	+
	*sfaS*	−	−	+	+
	*focF1C*	−	−	+	−
	*m* (type I)	+	+	+	+
	*afa*	−	−	−	−
	*nfaE*	−	−	−	−
	*gafD*	−	−	−	−
Capsule	*kpsMTII*	+	+	+	+
	*kpsMTIII*	−	−	−	−
	*rfc*	−	−	−	−
Iron acquisition system	*ent*	+	+	+	+
	*iro*	+	−	+	+
	*chu*	+	+	+	+
	*sit*	+	+	+	+
	*fyuA*	+	+	+	+
	*iutA*	−	−	+	−
Toxin	*cnf1*	−	−	−	+
	*cdt*	−	−	−	−
	*cvaC*	+	−	−	−
	*hlyA*	−	−	+	+
	*vat*	+	+	+	+
	*sat*	−	−	+	−
Invasin	*ibeA*	−	+	−	+
Pathogenicity-associated island	PAI	+	+	+	+
Resistance to serum	*traT*	−	−	−	+
Evasion of immune response	*tcpC*	−	−	+	−
Phylogroup		B2	B2	B2	B2
Biofilm production		Strong	Strong	ND^*a*^	ND

a ND, not determined.

### LF82 and EC73 strains adhere to, invade, and survive within RWPE-1 cell monolayers.

**FIG 1:**
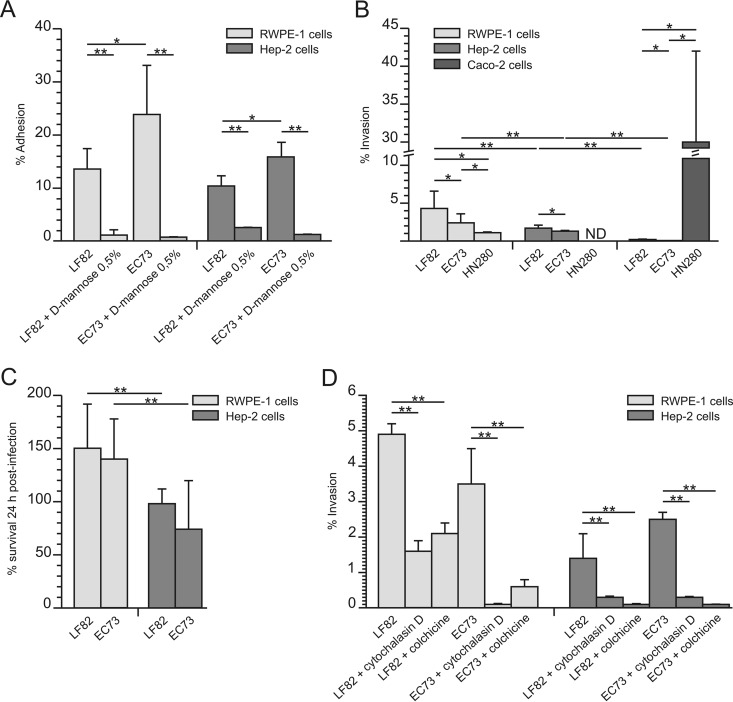
LF82 and EC73 adhesion (A), invasion (B), and ability to survive within infected cells (C) and role of host cell actin polymerization and microtubule in the invasion process (D). Data are expressed as means ± standard deviations from at least three independent experiments performed in triplicate. Asterisks indicate statistically significant differences at *P* ≤ 0.05 by ANOVA with *post hoc* unpaired (*) or paired (**) Student's *t* test. ND, not determined.

The adhesive, invasive, and intracellular survival abilities of LF82 and EC73 were assayed by infecting cell monolayers, as described in Materials and Methods. As for adhesiveness, both LF82 and EC73 were found to efficiently adhere to RWPE-1 and HEp-2 cells. Moreover, experiments performed in the presence of 0.5% d-mannose significantly reduced bacterial adhesion, confirming the key role of type 1 pili in this phenomenon (Fig. 1A). The gentamicin protection assay was used to assess the ability of the strains to invade and to survive within the infected cells. As shown in Fig. 1, compared to the noninvasive control E. coli K-12 strain MG1655, LF82 was able to invade (albeit to different extents) RWPE-1, HEp-2, and Caco-2 cells. Concerning EC73, the results obtained showed that it is able to efficiently invade RWPE-1 and HEp-2 cells but not Caco-2 cells. On the other hand, the EIEC strain HN280 invaded with high efficiency Caco-2 cells, while a low efficiency of invasion in prostate cells was observed (Fig. 1B), confirming the characteristics of a true gut pathogen. Concerning intracellular survival, LF82 and EC73 were able to survive and replicate within RWPE-1 cells, while they showed reduced survival within HEp-2 cells (Fig. 1C). Taken together, these results confirmed the pathobiont nature of LF82, which is able to invade and survive within prostate cells and possibly cause urogenital infections.

### Actin polymerization and microtubule recruitment are involved in bacterial uptake.

To evaluate the role of actin microfilaments and microtubules in bacterial uptake, cell monolayers were treated with either cytochalasin D or colchicine, as described in Materials and Methods. The addition of either cytochalasin D or colchicine to cell monolayers markedly inhibited bacterial entry (Fig. 1D). As a control, cells were infected with the same strains in the absence of the inhibitors (100%). These results clearly indicated that the activity of actin microfilaments and microtubules was highly required for bacterial uptake of both LF82 and EC73.

### Microscopic studies confirmed the intracellular localization of LF82 and EC73 strains.

**FIG 2:**
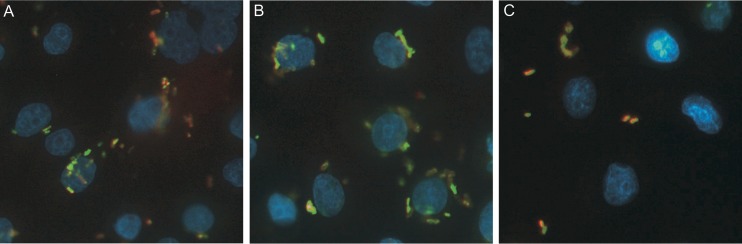
Immunofluorescence staining of RWPE-1 cell monolayers 24 h postinfection. (A) LF82; (B) EC73; (C) noninvasive control strain MG1655. Intracellular bacteria stained green, while extracellular bacteria appear orange/yellow. Magnification, 400×.

**FIG 3:**
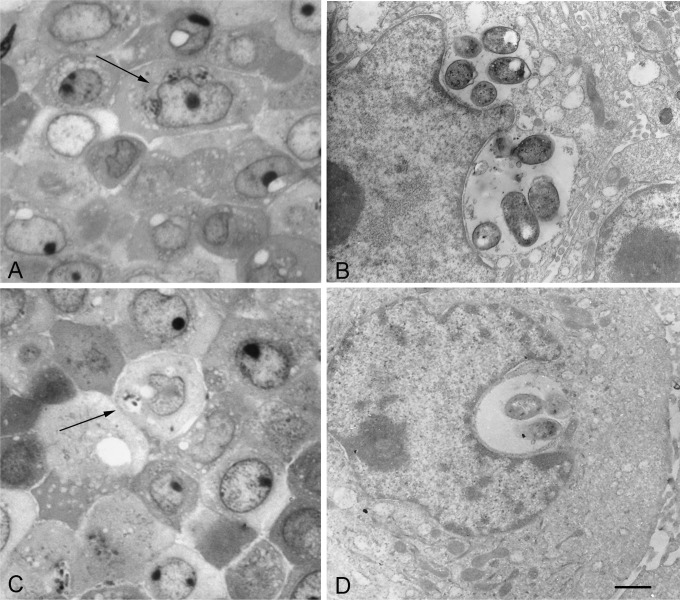
Light and electron microscopy of Epon-embedded RWPE-1 cells infected with LF82 (C and D) or EC73 (A and B) for 24 h. Groups of bacteria are frequently observed to occupy cytoplasmic vacuoles, suggesting active proliferation and/or clustering. (A and C) Light micrograph of toluidine blue-stained thin sections. Arrows indicate cells with groups of bacteria within cytoplasmic compartments. (B and D) Electron micrographs of ultrathin sections. Bars: A and C, 8 μm; B and D, 0.9 μm.

The intracellular localization of E. coli strains LF82 and EC73 in the RWPE-1 cells was also confirmed by immunofluorescence and transmission electron microscopy. Immunofluorescence analysis showed that 24 h postinfection, both strains were localized within the cytoplasm of infected cells (Fig. 2). In these experiments (see Materials and Methods for details), extracellular bacteria stained orange/yellow, while intracellular LF82 and EC73 strains stained green (Fig. 2A and B). The noninvasive control strain MG1655 was found only extracellularly (Fig. 2C). Intracellular bacteria were also detected by light and transmission electron microscopy (Fig. 3). RWPE-1 cell monolayers were infected with strain LF82 (Fig. 3C and D) or EC73 (Fig. 3A and B) as described above, and extracellular bacteria were eliminated by the addition of 100 μg/ml of gentamicin. Twenty-four hours postinfection, both strains appeared within vacuoles in the cytoplasm of the prostate cell line. These results are in accordance with a previous study that reported LF82 within membrane-bounded vacuoles in some intestinal epithelial cells (30).

### LF82 activates MAPKs and NF-κB signaling pathways in the prostate RWPE-1 cell line.

**FIG 4:**
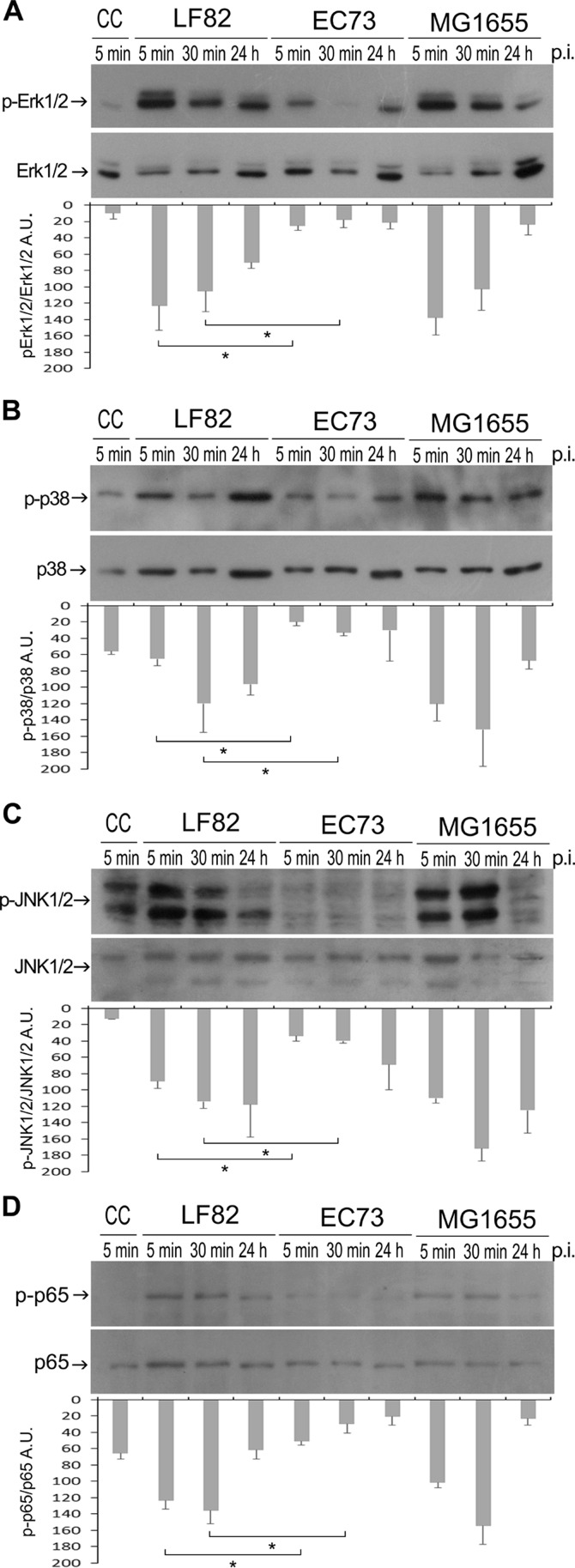
Phosphorylation of MAPKs and of the NF-κB p65 subunit induced by LF82 and EC73 in RWPE-1 cells. Shown are representative Western blots of cells infected for various lengths of time with different E. coli strains, as indicated (A, B, C and D). CC, uninfected cells. Stripped membranes were reprobed using antibodies anti-ERK1/2, anti-p38, anti-JNK1/2, and anti-p65 to control protein loading. The levels of phosphorylated proteins were quantified by densitometry (ImageJ software) and calculated as the ratios of phosphorylated to total kinases and phosphorylated to total p65. Data are expressed as arbitrary units (A.U.) and are means ± standard deviations from at least three independent experiments, in duplicate. Asterisks indicate statistically significant difference at *P* ≤ 0.05 by one-way ANOVA with *post hoc* unpaired Student's *t* test.

The mitogen-activated protein kinase (MAPK) family represents important signal transduction machinery and plays a prominent role in a wide range of cellular responses, including inflammation (40). Therefore, to determine the activation of MAPKs, RWPE-1 cell monolayers were infected (MOI of 10) with strains LF82, EC73, and MG1655, the latter a noninvasive strain known to be capable of triggering a MAPK cascade (41). At different time points after infection, whole-cell extracts were prepared and analyzed by Western blotting using specific antibodies (see Materials and Methods for details). Cells infected with strain LF82 as well as MG1655 displayed a prompt and dramatic increase in the amount of the phosphorylated forms of all MAPKs (Fig. 4A to C). Remarkably, cells infected with strain EC73 showed a basal level of MAPK phosphorylation throughout the time course experiment (Fig. 4A to C). The levels of activated NF-κB in the same whole-cell extracts were assessed. As shown in Fig. 4D, the phosphorylation of p65 was found to be much higher in LF82- and MG1655-infected samples than in EC73-infected samples. Overall, these results indicated that LF82 induces a stronger activation of both MAPKs and NF-κB pathways than EC73. This result is not surprising because it has been recently demonstrated that activation of NF-κB by LF82 is crucial for its intracellular survival (30).

### LF82 infection of RWPE-1 cells induces significantly higher release of IL-6 and IL-8 than EC73.

**FIG 5:**
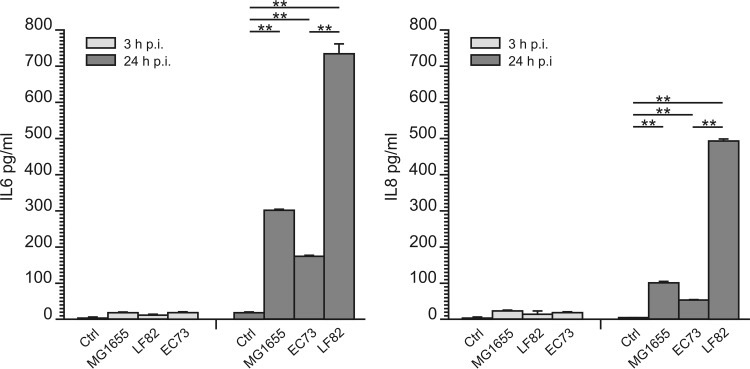
Levels of release of cytokines IL-6 and IL-8 in RWPE-1 cell monolayers infected with the E. coli LF82, EC73, and MG1655 strains (MOI of 10). Cytokines were measured 3 and 24 h postinfection by ELISA. Ctrl, control. Data are expressed as means ± standard deviations from at least three independent experiments. **, statistically significant difference at *P* ≤ 0.05 by repeated-measures ANOVA with *post hoc* paired Student's *t* test.

IL-6 and IL-8 represent two of the major cytokines produced by urinary epithelial cells following UPEC infection (42). To assess the ability of LF82 to induce release of proinflammatory cytokines, RWPE-1 cell monolayers were infected with E. coli strains LF82, EC73, and MG1655 as described above. Twenty-four hours postinfection, LF82 induced the release of an approximately 3-fold increase in IL-6 secretion and 5-fold increase in IL-8 secretion compared to EC73 (Fig. 5). This result is consistent with activation of MAPKs and NF-κB by LF82 (Fig. 4) and confirms secretion of proinflammatory cytokines upon LF82 infection of intestinal epithelial cells and in transgenic mice expressing human CEACAM (43).

## DISCUSSION

A subset of fecal E. coli strains, collectively called extraintestinal pathogenic E. coli (ExPEC), move out from the intestine gain access to extraintestinal niches, exploiting their ability to colonize and to cause disease (44). A variety of virulence factors are carried out by ExPEC strains; however, they display considerable genotypic and phenotypic diversity. For this reason, a generally accepted protocol to unambiguously differentiate ExPEC subtypes (namely UPEC, NMEC, and SEPEC) from commensals has not been established yet (45). Among ExPEC strains, UPEC strains are the main etiological agents of cystitis, acute/chronic prostatitis, and acute pyelonephritis (31, 46).

Adherent/invasive E. coli (AIEC) strains represent a group of pathogenic E. coli strains that have been associated with the initiation or maintenance of chronic inflammation in CD patients (7). AIEC strains do not harbor common virulence factors present in the enteropathogenic E. coli strains, and LF82 and NRG857c are two prototypes of this class of strains (13). Recently, on the basis of the available sequence data, a phylogenetic linkage between AIEC and ExPEC, in particular between strains able to cause UTI and NMEC, has been reported (7). Here, we first compared the virulence determinants shared by LF82 and UPEC strains EC73, CFT073, and UTI89 (Table 1). In accordance with previous reports (7), all strains were found to belong to the E. coli phylogroup B2 and to be biofilm producers. Biofilm production is known to be a key factor that facilitates bacterial colonization and persistence in the urinary and intestinal tracts, as well as resistance to the host innate immune response and to antibiotic therapy (45, 47). *In silico* virulence genotyping (Table 1) showed that LF82 shares with UPEC strains several relevant virulence factors: namely type 1 pili, group II capsule, the vacuolating autotransporter toxin, enterobactin, permease, and yersiniabactin receptor. These results clearly indicate that LF82 likely has the potential to colonize human sites different from the intestinal tract.

To assess whether LF82 might behave as an ExPEC strain, we used the human prostate RWPE-1 cell line, which shows many characteristics of the normal prostate epithelium, as an *in vitro* model to study host cell-bacterium interactions. Adhesion, invasion, and survival assays (Fig. 1) showed that LF82 and EC73 were able to adhere to, invade, and survive within prostate cells as well as within HEp-2 cells, chosen as a control. As previously reported using different cell models, the adhesiveness of AIEC and UPEC isolates is strongly inhibited by the presence of d-mannose (11, 48). Accordingly, we observed that the ability of LF82 and EC73 cells to adhere to the RWPE-1 and HEp-2 cell lines was significantly inhibited by d-mannose, indicating a pivotal role of type 1 pili in the adhesiveness to the prostate cell line. Furthermore, intracellular uptake of both strains was strongly inhibited in both cell lines by the addition of cytoskeleton inhibitors, indicating that invasion of prostate cells requires microtubule polymerization and actin recruitment. Moreover, to compare the invasive efficiency of a true intestinal pathogen (EIEC strain HN280) to that of LF82 and EC73, invasion assays were performed with both intestinal and prostate cells. Interestingly, while the EIEC strain efficiently invaded Caco-2 cells, it was almost unable to invade prostate cells (Fig. 1B). On the other hand, LF82 invaded prostate and Caco-2 cells, although to different extents. EC73 was able to invade prostate cells but failed to enter intestinal cells. Taken together these results confirmed the “pathobiont” nature of LF82.

Immunofluorescence and electron microscopy (Fig. 2 and 3) showed that LF82 and EC73 cells were intracellular and localized as multiple organisms within membrane-bound vacuoles of the prostate cell line, suggesting that they can replicate within these compartments. Next, the inflammatory response of infected prostate cells was evaluated by determining the phosphorylation of MAPKs and NF-κB factor 65 and the levels of secreted cytokines IL-6 and IL-8. The results obtained indicated that all strains were able to stimulate phosphorylation of MAPKs and NF-κB factor 65 already by 5 min postinfection. In particular, the highest phosphorylation was observed with cells infected with LF82 and with the K-12 noninvasive control strain MG1655. Compared to uninfected samples, RWPE-1 cells infected with LF82, EC73, and MG1655 released increased amounts of IL-6 and IL-8 24 h postinfection (Fig. 5), but while LF82 induced marked production of both cytokines, lower levels were detected in cells infected with EC73 and MG1655. As for EC73, these data are consistent with previous reports (22, 27, 42) indicating that some UPEC strains, in order to persist within host urothelial cells, can adopt different strategies leading to silencing of Toll-like receptor 4 (TLR4) signaling and NF-κB activity, to modulate the release of proinflammatory cytokines. This finding led us to speculate that EC73 is likely well adapted to persist in infected urothelial and prostate cells. On the other hand, different from EC73, AIEC strain LF82 strongly activates NF-κB signaling pathways and IL-6 and IL-8 secretion. Recently, it has been reported that LF82 utilizes two complementary mechanisms to survive within the intestinal mucosa: one is evasion of inflammasome activation and the other is activation of the NF-κB pathways. Furthermore, it has also been shown that the blockade of LF82-induced NF-κB activation leads to a massive epithelial cell apoptosis (30). Interestingly, by activating NF-κB, LF82 could likely protect cells from apoptosis, inducing favorable conditions to survive within prostate cells. (LF82 can survive in RWPE-1 cells 10 days postinfection without inducing any sign of apoptosis [data not shown].) Starting from our data, a further study of the mechanisms underlying LF82 persistence in the prostate cells could have significant implications for understanding how in prostate cells, AIEC can cause an acute infection mediated by massive cytokine production, whereas a UPEC strain causing recurrent UTIs can establish persistent colonization through the induction of low levels of inflammatory mediators. In conclusion, the present work indicates that AIEC strains may well behave like ExPEC strains, which moving from the intestinal tract, may cause extraintestinal infections. The present data on human prostate cells will hopefully encourage medical attention to evaluate the actual impact of AIEC infections in prostate/genitourinary tract samples. In parallel, animal models can be envisaged for experimentation on the detailed characteristics of such extraintestinal colonization by different strains of E. coli.
